# C-reactive protein, established risk factors and social inequalities in cardiovascular disease – the significance of absolute versus relative measures of disease

**DOI:** 10.1186/1471-2458-8-189

**Published:** 2008-06-02

**Authors:** Maria Rosvall, Gunnar Engström, Göran Berglund, Bo Hedblad

**Affiliations:** 1 Social Epidemiology, Department of Clinical Sciences in Malmö, Lund University, Malmö University Hospital, Malmö, Sweden; 2 Epidemiological Research Group, Department of Clinical Sciences in Malmö, Lund University, Malmö University Hospital, Malmö, Sweden; 3 Clinic of Internal Medicine, Department of Clinical Sciences in Malmö, Lund University, Malmö University Hospital, Malmö, Sweden

## Abstract

**Background:**

The widespread use of relative scales in socioepidemiological studies has recently been criticized. The criticism is based mainly on the fact that the importance of different risk factors in explaining social inequalities in cardiovascular disease (CVD) varies, depending on which scale is used to measure social inequalities. The present study examines the importance of established risk factors, as opposed to low-grade inflammation, in explaining socioeconomic differences in the incidence of CVD, using both relative and absolute scales.

**Methods:**

We obtained information on socioeconomic position (SEP), established risk factors (smoking, hypertension, and hyperlipidemia), and low-grade inflammation as measured by high-sensitive (hs) C-reactive protein (CRP) levels, in 4,268 Swedish men and women who participated in the Malmö Diet and Cancer Study (MDCS). Data on first cardiovascular events, i.e., stroke or coronary event (CE), was collected from regional and national registers. Social inequalities were measured in relative terms, i.e., as ratios between incidence rates in groups with lower and higher SEP, and also in absolute terms, i.e., as the absolute difference in incidence rates in groups with lower and higher SEP.

**Results:**

Those with low SEP had a higher risk of future CVD. Adjustment for risk factors resulted in a rather small reduction in the relative socioeconomic gradient, namely 8% for CRP (≥ 3 mg/L) and 21% for established risk factors taken together. However, there was a reduction of 18% in the absolute socioeconomic gradient when looking at subjects with CRP-levels < 3 mg/L, and of 69% when looking at a low-risk population with no smoking, hypertension, or hyperlipidemia.

**Conclusion:**

C-reactive protein and established risk factors all contribute to socioeconomic differences in CVD. However, conclusions on the importance of "modern" risk factors (here, CRP), as opposed to established risk factors, in the association between SEP and CVD depend on the scale on which social inequalities are measured. The one-sided use of the relative scale, without including a background of absolute levels of disease, and of what causes disease, can consequently prevent efforts to reduce established risk factors by giving priority to research and preventive programs looking in new directions.

## Background

The associations between socioeconomic position (SEP) and cardiovascular disease (CVD) are well documented and relatively undisputed [1-3]. In the search for causal explanations of socioeconomic differences in cardiovascular diseases, it has been shown repeatedly that established risk factors such as smoking, physical inactivity, blood pressure, total-cholesterol, body mass index, and blood glucose, account for less than 50% of the socioeconomic differences in coronary heart disease (CHD) [4,5]. Much less is known about the reasons for the differences in cerebrovascular disease [6]. Research into causal explanations of socioeconomic differences in CVD has most often used a relative scale, and it has been argued that relative measures are more appropriate for etiological investigations than absolute measures [7]. The increasing awareness of the limitations of established risk factors in explaining socioeconomic differences in CVD has stimulated research in new directions and has revitalized old ideas. One example of this is the increasing recognition of an inflammatory component of atherogenesis [8,9], where major acute-phase-proteins such as high sensitive (hs) C-reactive protein (CRP) and fibrinogen have been found to predict acute cardiovascular events [8]. Thus, inflammation could in theory be another factor that might explain the social gradient in CVD. However, the notion that established risk factors have only modest importance in explaining socioeconomic differences in CVD, followed by a search for undiscovered risk factors, has been criticized [10,11]. In a recent study by Lynch et al. [11], it is well illustrated that the conclusions on the importance of various risk factors heavily depend on the scale on which social inequalities are measured. The authors criticize the one-sided use of the relative scale over the past two decades, and they want to make visible that an absolute risk approach focuses attention on those risk factors that cause most cases of disease. The consistent use of the relative scale has important implications for the choice of preventive strategies, in that the quest for new factors may be at the expense of trying to reduce the levels of established risk factors that explain most cases of CVD.

The aim of the present study was to replicate the analyses by Lynch and co-authors [11] on the importance of various risk factors in explaining socioeconomic differences in CVD, this time in a Swedish context using similar methodology. We wanted to examine the importance of established risk factors, as opposed to low-grade inflammation as measured by CRP levels, in explaining socioeconomic differences in the incidence of CVD. However, instead of restricting the analyses to coronary heart disease as in the study by Lynch et al., we wanted to use a broader measure of CVD, including both CE and stroke. In the analyses, social inequalities were measured in relative terms, i.e. as ratios between incidence rates in lower and higher SEP groups, and also in absolute terms, i.e. the absolute difference in incidence rates in lower and higher SEP groups.

## Methods

### Study population

The subjects in this study constituted a sub-cohort of the large, population-based Malmö Diet and Cancer Study (MDCS) cohort [12,13]. A random fifty percent sample of those born between 1926 and 1945 who entered the MDCS between October 1991 and February 1994 (n = 12,445) were invited to take part in a study on the epidemiology of carotid artery disease [14]. We included those individuals who had accepted the invitation to join the carotid artery disease study and who had completed a self-administered questionnaire with questions on social and psychosocial factors, which was completed as part of the baseline examination (n = 4,884) [15]. Subjects were considered to have CVD if they had been treated for myocardial infarction and/or stroke according to the national or regional myocardial infarction register or stroke register. Subjects with known CVD (87 men and 29 women) were excluded from the analyses. Another 344 individuals were excluded because of missing laboratory results, 150 individuals because of missing data on CRP, and 6 individuals because of missing data on educational level. The remaining 4,268 subjects, 2,501 women and 1,767 men aged 46–68 years, constituted the study population.

The study was approved by the Ethics Committee at Lund University. All participants gave their informed consent.

#### Cardiovascular risk factors

Risk factors were estimated through laboratory tests, examination at baseline and through the questionnaire administered at the baseline visit. Details of assessment procedures regarding smoking habits (never, former, and current smoker), measurements of supine blood pressure (mm Hg), low-density lipoprotein (LDL), high-density lipoprotein (HDL), diabetes, use of blood pressure lowering medication, and treatment for hyperlipidemia have been reported previously [15]. Subjects were classified as having hyperlipidemia if they used medication against hyperlipidemia or if they had hypercholesterolemia (total cholesterol ≥ 6.5 mmol/L) and/or hypertriglyceridemia (defined as triglyceride levels > 2.3 mmol/L according to the Swedish guidelines for treatment of hyperlipidemia) [16]. Prevalent hypertension was defined as systolic blood pressure of 160 mm Hg or more, a diastolic blood pressure of 90 mm Hg or more, or self-reported use of antihypertensive medication. Subjects were classified as having diabetes mellitus if they reported the diagnosis in the questionnaire, used anti-diabetic medication or had a fasting whole venous blood glucose level of ≥ 6.1 mmol/L. The analysis of CRP was done from frozen plasma samples gathered at the baseline examination using the Tina-quant^® ^CRP latex high sensitivity assay (Roche Diagnostics, Basel, Switzerland) on an ADVIA^® ^1650 Chemistry System (Bayer Healthcare, NY, USA). Study samples were analyzed as discrete samples and results were read in 6-second intervals for a time period of one minute following incubation for 5 minutes. The mean value of these measurements was the result reported. The average coefficient of variation (CV) has in earlier studies on the MDCS been documented as 4.59% [17].

#### Measurement of incident events

A cardiovascular event was defined as first CE or first stroke, whichever came first.

For CE, each individual was followed until December 31, 2001, date of first CE, or death. Information on morbidity and mortality in the MDCS was obtained by record linkage with the National Inpatient Register (Swedish Board on Health and Welfare), the Swedish Causes of Death Register [18], and the Malmö Myocardial Infarction Register [19]. Underlying causes of death and hospitalization diagnosis, respectively, were coded in accordance with the ninth version of the International Classification of Diseases [20]. A coronary event was defined as fatal or non-fatal myocardial infarction (code 410), or death caused by ischemic heart disease (codes 412 and 414).

Record linkage with the Stroke Register of Malmö (STROMA) gave information on morbidity and mortality from stroke in the MDCS [21,22]. The National Inpatient Register was used to retrieve information on cases who had moved away from Malmö during follow-up. Information on case retrieval, validity, and ascertainment of cases in the MDCS has been described in detail previously [23]. Briefly, all cases were followed from baseline examination, until death, or December 31, 2001. Stroke was defined as rapidly developing clinical signs of local or global loss of cerebral functioning lasting > 24 h (or leading to death before then). Classification as subarachnoid hemorrhage (ICD-9 code 430) or intracerebral hemorrhage (ICD-9 code 431) required verification by computed tomography (CT) and/or autopsy. Cerebral infarction (ICD-9 code 434) was diagnosed when CT or autopsy could verify the infarction and/or exclude hemorrhage and nonvascular disease. In subjects with more than one stroke event, only the first event was used for the analyses.

#### Measurement of educational level

Information on educational level was assessed using a self-administered questionnaire. Educational level was classified into three categories based on the length of educational achievement: (1) low educational level, i.e. 8 years of education or less, (2) intermediate educational level, i.e. 9–12 years of education, and (3) high educational level, i.e. more than 12 years of education [15].

#### Statistical methods

Relative differences in mortality by educational level were assessed by crude and also age-and sex-adjusted hazard ratios (HR) in a Cox proportional hazards model. We calculated absolute rate differences (ARD) in incidence of CVD between educational groups (expressed as cases per 100,000 person-years at risk). Age- and sex-adjusted incidence rates of first cardiovascular event according to educational level, were analyzed by a direct standardization with equal weights. The analyses were performed: (a) on the whole population, (b) on a low-risk population of non-smokers without hypertension, and hyperlipidemia and (c) on a population with CRP levels below 3 mg/L. Furthermore, similar analyses were performed on a low-risk population also excluding those with diabetes mellitus and on subjects with CRP levels below 1 mg/L.

## Results

### Baseline characteristics of the study population

Details of the study population are given in Table 1. Cardiovascular event cases showed a pattern of risk factors that was generally less favorable than that of non-cases. For example, among the cases 33% had CRP levels exceeding 3 mg/L, 40.1% were current smokers, 55.1% had hyperlipidemia, 62.8% had hypertension and 12.5% had diabetes. The corresponding percentages among the non-cases were 22.4%, 22.1%, 46.4%, 39.3% and 4.6%, respectively. Altogether, 92% of the cases had at least one established risk factor (i.e. hypertension, hypercholesterolemia or smoking), 41% had two risk factors, and 9% had all three risk factors. The corresponding proportions among the non-cases were 70%, 29% and 3%. The cases had a significantly higher proportion of subjects with low educational attainment than the non-cases.

**Table 1:** Age-and sex-adjusted means and prevalences of educational level and cardiovascular risk factors by cardiovascular event status

	Cardiovascular event‡	No cardiovascular event
	
	(n = 196)	(n = 4,072)
Age, years	60.5*	57.1
Male (%)	59.3*	39.9
Current smoking (%)	40.1*	22.1
Former smoking (%)	31.0	34.3
Hyperlipidemia (%)†	55.1*	46.4
Hypertension (%)†	62.8*	39.3
CRP above 3 mg/L (%)	33.0*	22.4
Diabetes mellitus (%)	12.5*	4.6
Educational level		
8 years or less	59.8*	46.7
9 to 12 years	30.7	34.2
13 years or more	8.6*	18.9

* P-values are given for the difference in risk factor levels between those with and those without a cardiovascular event; *p < 0.05.

† Hyperlipidemia is defined as use of medication against hyperlipidemia, hypercholesterolemia (total-cholesterol > 6.5 mmol/L) and/or hypertriglyceridemia defined as triglycerides > 2.3 mmol/L; Hypertension was defined as systolic bloodpressure of 160 mm Hg or more, a diastolic bloodpressure of 90 mm Hg or more, or self-reported use of antihypertensive medication.

‡ A cardiovascular event was defined as first coronary event or first stroke, whichever came first.

### Educational level and first cardiovascular event in the whole population

As can be seen in Table 2, in the crude model, those with low educational attainment had a more than three-times higher hazard of incident CVD than those with high educational attainment. The corresponding hazard in those with an intermediate level of education was twofold. The absolute educational differences in incidence rates were 523 per 100,000 person-years when comparing the lowest and the highest educational groups and 216 per 100,000 person-years when comparing the groups with intermediate and highest levels of education. Similar patterns of associations were seen in the age- and sex-adjusted model, although the relative and absolute socioeconomic differences were smaller than in the crude model.

**Table 2:** Relation between educational level and incident cases of first cardiovascular event (stroke or coronary event) in the whole population.

			Crude model			Age- and sex-adjusted model		
			
Educational level	N (%)	No. of cases	Incidence rate (per 100 000)	Hazard ratio (95% CI)†	Absolute difference	Incidence rate (per 100 000)	Hazard ratio (95% CI) †	Absolute difference
8 years of education or less	1,961	124	748	3.4 (2.0, 5.6)	523	683	2.8 (1.6, 4.7)	433
9 to 12 years of education	1,483	56	441	2.0 (1.1, 3.4)	216	456	1.9 (1.1, 3.4)	206
More than 12 years of education*	824	16	225	1.0	0	250	1.0	0
								
Total	4,268	196	539			531		

*Reference category

†CI; confidence interval

### CRP and first cardiovascular event in the whole population

CRP was associated with an increased risk of future cardiovascular events even after adjustment for cardiovascular risk factors, i.e. age, sex, current smoking, hypertension, hyperlipidemia, diabetes, and BMI, where those with CRP levels above 3 mg/L had an HR of 1.6 (95% confidence interval, CI: 1.1–2.4) compared to those with CRP levels below 1 mg/L. Those with CRP levels between 1 and 3 mg/L also showed an increased risk of a future cardiovascular event, with an HR of 1.4 (95% CI: 1.0–2.1).

### Educational level and first cardiovascular event in a population with CRP levels below 3 mg/L

In subjects with CRP levels below 3 mg/L, the group with a low level of education had an HR of 3.2 (95% CI: 1.7–6.2) and the group with an intermediate level of education had an HR of 2.2 (95% CI: 1.1–4.2) compared to the group with a high educational level (Table 3). This corresponds to a reduction of approximately 8% and an increase of approximately 20% in the relative inequalities seen between the low and intermediate educational level groups, respectively, and the high educational level group, compared to when looking at the relative differences in the whole population. The absolute educational differences were 429 per 100,000 person-years and 223 per 100,000 person-years for the groups with low and intermediate educational levels, respectively, relative to the group with high educational level. Similar patterns of associations were seen in the age- and sex-adjusted model, although with smaller relative and absolute socioeconomic differences than for the crude model.

**Table 3:** Relation between educational level and incident cases of first cardiovascular event (stroke or coronary event) in a population with CRP-levels below 3.

			Crude model			Age- and sex-adjusted model		
			
Educational level	N (%)	No. of cases	Incidence rate (per 100 000)	Hazard ratio (95% CI) †	Absolute difference	Incidence rate (per 100 000)	Hazard ratio (95% CI)†	Absolute Difference
8 years of education or less	1,443	76	619	3.2 (1.7, 6.2)	429	573	2.7 (1.4, 5.1)	358
9 to12 years of education	1,185	42	413	2.2 (1.1, 4.2)	223	443	2.2 (1.1, 4.3)	228
More than 12 years of education*	668	11	190	1.0	0	215	1.0	0
								
Total	3,296	129	457			467		

*Reference category

†CI; confidence interval

**Figure 1:**
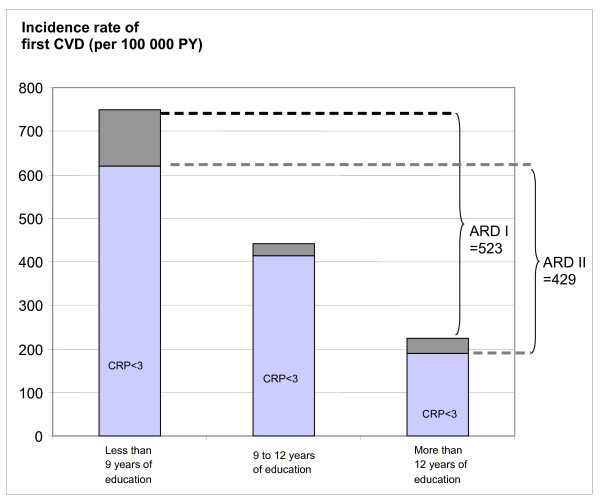
**Crude incidence rates of first cardiovascular event (i.e. stroke or coronary event) by educational level in the whole population (shown as dark grey and light grey bars) and in a population with CRP below 3 mg/L (shown as light grey bars).** ARD I stands for absolute difference in incidence rate between those with less than 9 years of education and those with more than 12 years of education in the whole population. ARD II stands for the corresponding difference in a population with CRP-levels below 3 mg/L.

As seen in Figure 1, the difference in crude incidence rates between the group with low educational level and the group with high educational level was 523 per 100,000 person-years in the whole study population and 429 per 100,000 person-years in subjects with CRP levels below 3 mg/L. This gives a reduction in the excess risk between the low and high educational level groups of 18%, i.e. (523-429/523) * 100 compared to when looking at the whole study population. The corresponding negative reduction (i.e. increase) in excess risk between the groups with intermediate and high educational levels was 3%, i.e. (216-223/216) * 100. In the age- and sex-adjusted model (not shown in the figure), the reduction in excess risk between the groups with low and high educational level was 17%, i.e. (433-358/433) * 100.

### Educational level and first cardiovascular event in a population with CRP levels below 1 mg/L

In subjects with CRP levels below 1 mg/L (n = 1,797; cases = 51), the group with low educational level had an HR of 4.2 (95% CI: 1.5–12.1) compared to the group with high educational level (data not shown). This corresponds to an approximately 33% increase in the relative inequalities seen between the low educational level group and the high educational level group compared to when using the whole population. The crude incidence rates in the groups with high, intermediate, and low educational levels were 115, 292, and 484 per 100,000 person-years, respectively. The absolute educational differences were 369 and 177 per 100,000 person-years, for the low and intermediate educational level groups, respectively, and the high educational level group. This gives a reduction in the excess risk between the groups with low and high educational levels of 29%, i.e. (523-369/523) * 100 compared to the whole population. The corresponding reduction in excess risk between the groups with intermediate and high educational levels was 18%, i.e. (216-177/216) * 100.

### Educational level and first cardiovascular event in a low risk population

In a low-risk population (i.e. no hypertension, no hypercholesterolemia, and no current smoking), the group with low educational level had an HR of 2.9 (95% CI: 0.6–13.2) and the group with intermediate educational level had an HR of 1.8 (95% CI: 0.4–8.9) compared to the high educational level group (Table 4). This corresponds to reductions of approximately 21% and 20% in the relative inequalities seen between the low and intermediate educational level groups, respectively, and the high educational level group compared to when using the whole study population. The absolute educational differences were 163 per 100,000 person-years and 70 per 100,000 person-years, respectively, for the groups with low and intermediate educational levels, respectively, compared to the high educational level group. Similar patterns of associations were seen in the age- and sex-adjusted model, although with smaller relative and absolute socioeconomic differences than for the crude model.

**Table 4:** Relation between educational level and incident cases of first cardiovascular event (stroke or coronary event) in a low-risk population‡.

			Crude model			Age- and sex-adjusted model		
			
Educational level	N (%)	No. of cases	Incidence rate (per 100 000)	Hazard ratio (95% CI) †	Absolute difference	Incidence rate (per 100 000)	Hazard ratio (95% CI) †	Absolute difference
Less than 9 years of education	408	9	253	2.9 (0.6, 13.2)	163	229	2.0 (0.4, 9.1)	93
9 to 12 years of education	428	6	160	1.8 (0.4, 8.9)	70	182	1.5 (0.3, 7.6)	46
More than 12 years of education*	256	2	90	1.0	0	136	1.0	0
								
Total	1,092	17	151			193		

*Reference category

†CI; confidence interval

‡Low risk population includes those with no current smoking, no hypertension (i.e., systolic blood pressure above 160 mm Hg and/or diastolic blood pressure above 90 mm Hg and/or treatment for hypertension), and no hyperlipidemia (i.e., total-cholesterol > = 6.5 mmol/L and/or triglycerides > 2.3 mmol/L and/or treatment for hyperlipidemia).

**Figure 2:**
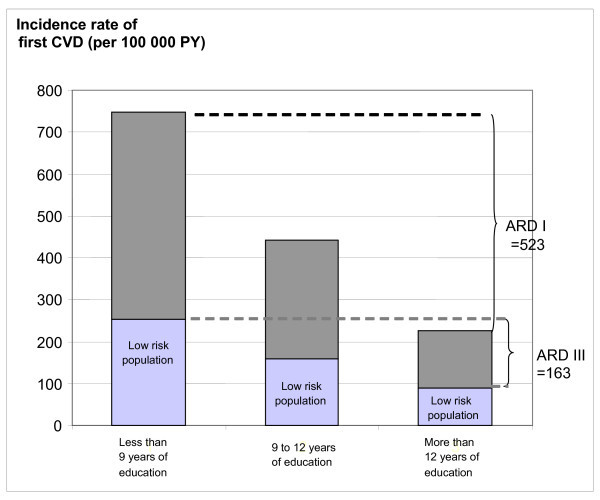
**Crude incidence rates of first cardiovascular event (i.e. stroke or coronary event) by educational level in the whole population (shown as dark grey and light grey bars) and in a low-risk population (i.e., with no hypertension, no hypercholesterolemia and no current smoking) (shown as light grey bars).** ARD I stands for absolute difference in incidence rate between those with less than 9 years of education and those with more than 12 years of education in the whole population. ARD III stands for the corresponding difference in the low-risk population.

As seen in Figure 2, the difference in crude incidence rates between the low educational level group and the high educational level group was 523 per 100,000 person-years in the whole study population and 163 per 100,000 person-years in the low-risk population. This gives a reduction in the excess risk between the groups with low and high educational levels of 69%, i.e. (523-163/523) * 100 compared to when looking at the whole study population. The corresponding reduction in excess risk between the groups with intermediate and high educational levels was 67%, i.e. (216-70/216) * 100. In the age- and sex-adjusted model (not shown in the figure), the reduction in excess risk between the low and high educational level groups was 78%, i.e. (433-93/433) * 100 and for the intermediate educational level group it was 78%, i.e. (206-46/206) * 100.

### Educational level and first cardiovascular event in a low-risk population, also adding absence of diabetes

If one also adds absence of diabetes when defining the low-risk population (n = 1,064; cases = 15), the group with a low educational level had an HR of 2.4 (95% CI: 0.4–9.1) and the group with an intermediate level of education had an HR of 1.8 (95% CI: 0.5–10.9) compared to the group with a high level of education (data not shown). This corresponds to reductions of approximately 41% and 20% in the relative inequalities seen between the low and intermediate educational level groups, respectively, and the high educational level group compared to when using the whole study population. The crude incidence rates in the groups with high, intermediate, and low educational levels were 90, 164, and 204 per 100,000 person-years, respectively. The absolute educational differences were 114 per 100,000 person-years and 74 per 100,000 person-years, between the low and intermediate educational level groups, respectively, and the high educational level group. This gives a reduction in the excess risk between the groups with low and high educational levels of 78%, i.e. (523-114/523) * 100 compared to when looking at the whole study population. The corresponding reduction in excess risk between the groups with intermediate and high educational levels was 66%, i.e. (216-74/216) * 100.

## Discussion

As has been shown in earlier studies [1-3,24], adjustment for cardiovascular risk factors resulted in a rather small reduction in the relative educational gradient in risk of future CVD, i.e. 8% for CRP and 21% for established risk factors taken together (current smoking, hypertension and hyperlipidemia). When considering absolute educational differences, however, there was a reduction in the absolute gradient of 18% for CRP levels below 3 mg/L and of 69% for the established risk factors taken together. The reduction in the latter group was even more pronounced if also excluding subjects with diabetes from the low-risk population. Thus, conclusions on the importance of "modern" and established risk factors depend on which scale one measures socioeconomic differences in morbidity and mortality. This has important implications for public health strategies and the preventive potential of acting on reducing established risk factors rather than "modern" risk factors. Our results are similar to the results from a recent population-based study of 2,682 men in eastern Finland [11]. For highly prevalent risk factors with strong associations with CVD, as in the case of established risk factors, there were strong reductions in absolute risk in each educational category after stratification (i.e., looking at a low-risk population without smoking, hypertension or hyperlipidemia). There were, however, only rather small changes in the relative gradient, while for the "modern" risk factor (here CRP) there were rather small changes in the relative gradient and only small reductions in absolute risk after stratification (i.e., looking at a population with CRP-levels below 3 mg/L). It is well known that relative measures are cumbersome when it comes to making comparisons, e.g. between age groups, sexes, countries, or populations [25]. Furthermore, when evaluating the importance of mediating factors or confounders, relative comparisons can be misleading as the effect of a given association depends heavily on the distribution of the exposure variable in the different categories of the mediating/confounding factor [11].

Primary and secondary prevention of CVD has mainly included prevention of established risk factors, and the risk of disease has decreased by 40% since the mid-1970s [26]. Regarding social inequalities in cardiovascular health, however, there have been similar or even widening relative social inequalities over time [27,28]. Those who have benefited most from these interventions appear to have been individuals with a higher level of education, and the relative inequalities are thus widening [27]. If one considers the absolute levels, however, there has been a decrease of CVD in all socioeconomic groups over time. Thus, the conclusion about the importance of established risk factors in explaining the socioeconomic differences depends on the scale on which one measures social inequalities. These are important issues that affect opinions about where to place the effort in preventive programs – on established risk factors or on modern risk factors. It has recently been suggested that even though the use of relative measures is helpful in the search for mechanistic links between SEP and CVD, it is important to interpret the findings against a background of absolute levels of disease, and on what causes disease [11], in order to better interpret the effect of a certain factor on a given association.

In recent years, there has been increasing recognition of the inflammatory component of atherogenesis. Levels of major acute-phase-proteins such as CRP and fibrinogen have been found to predict acute cardiovascular events in prospective studies [8]. The inflammatory marker CRP has been found to be associated with the presence of atherosclerosis and to double the risk of future cardiovascular events when exceeding 3 mg/L [8]. However, there have also been studies that have shown that the usefulness of CRP in prediction beyond that of established risk factors is small [29,30]. Furthermore, previous studies have shown an inverse association between SEP and levels of CRP, serum amyloid A (SAA), and fibrinogen [31-37]. In a recent study on the MDCS cohort, we found that low SEP was strongly associated with CRP levels, independently of potential mediating factors e.g., smoking and factors involved in the metabolic syndrome. Furthermore, CRP levels were found to be associated with the extent of carotid atherosclerosis. However, there was only a minor attenuation of the relative SEP gradient in carotid atherosclerosis after adjustment for CRP [9]. Similarly, in the present study there was only a small reduction in the relative educational gradient in the risk of cardiovascular events, and also a small reduction in the absolute educational gradient after stratification for CRP below 3 mg/L. It should be noted that this was for the unadjusted measure of CRP, i.e. some of these reductions in the gradients were due to established risk factors known to be associated with CRP such as smoking, hypertension, and BMI [8]. It is difficult to directly compare the effects of CRP and established risk factors for several reasons. Firstly, stratification of established risk factors also results in a reduction in mean CRP levels and stratification for CRP results in lower levels of the established risk factors. The absolute risk reductions are thus influenced by other risk factors that correlate with the risk factors for which the analysis was stratified. Secondly, some of the effects of CRP may be mediated through the development of other risk factors. Longitudinal studies have shown that low-grade inflammation is associated with the development of hypertension, diabetes and large weight gain [38-40]. Similarly, the effects of the established risk factors could be mediated through their proinflammatory effects. Thirdly, stratification of CRP resulted in a larger group (n = 3,296) than the group without major risk factors (n = 1,092), and the low-risk group was therefore a more selected group with lower cardiovascular risk.

Certain methodological issues must to be addressed. Firstly, misclassification of endpoint is a possible cause of bias. However, about 95% of the cases of stroke were confirmed by CT and/or autopsy [23]. Vital status of all individuals at the end of follow-up was updated by data linkage with the regional stroke register in Malmö, and with the regional and national myocardial infarction register [19,21]. The STROMA is a population-based register that is known to be better than hospital-based registers with regard to coverage and potential selection bias [41]. The completeness and validity of the national myocardial infarction register and STROMA has been documented in several other studies [22,42]. The proportion of non-hospitalized cases is very small in Sweden. There is no reason to believe that incomplete retrieval of cases biased the results.

Misclassification of exposure is a potential cause of bias. Educational level, usually attained in early adulthood, should not to be influenced by clinical atherosclerotic disease, which occurs later in life. The use of education as a marker of SEP has been shown to be reliable, to have a low non-response rate and, as it is usually attained in early adulthood, not to be subject to reverse causation [43,44]. However, education might be a problematic indicator in a study covering a wide range of age cohorts, i.e. social and economical values might differ between various birth cohorts [45]. Other studies have shown a weaker association between SEP and various disease outcomes in groups close to retirement age, which might be partly attributable to survivor bias [46]. However, an earlier study on the MDCS showed a similar association between educational level and risk of future coronary events in subjects below 60 years of age and in the whole population [47].

Furthermore, misclassification of mediating factors is another potential source of bias. CRP has been suggested to be a good indicator of low-grade inflammation since the levels appear to be reasonably stable over time, with little seasonal variation [48,49]. While some studies have shown signs of diurnal variation [49], others have not [50]. CRP has been shown to be related to future cardiovascular events [8], and this was also true in our study, even after adjustment for potential confounders such as smoking, hypertension, hyperlipidemia, BMI, and diabetes mellitus. The definition of a low-risk population in the present study was based on the three major cardiovascular risk factors, i.e., current smoking, hypertension and hyperlipidemia. The definition of hypertension using a systolic blood pressure exceeding 160 mm Hg is in accordance with the national guidelines at the time of baseline investigation in the early 1990s.

This study is based on a community-based sample of the general population, which makes it less sensitive to selection bias than samples based on workplace or populations in clinical settings. However, excluding subjects with known CVD together with the fact that people who participate in public health surveys are generally healthier than the non-participants might lead to an underestimation of the true associations between the measures of education and incident cardiovascular events in our study.

## Conclusion

As shown in earlier studies, adjustment for cardiovascular risk factors resulted in a rather small reduction in the relative educational gradient in incidence of CVD, i.e. 8% for CRP and 21% for established risk factors taken together. However, in terms of absolute educational differences in CVD incidence, the risk reductions were 18% and 69%, respectively. Thus, the conclusion on the importance of a risk factor depends on which scale one measures social inequalities. In the case of highly prevalent risk factors with strong associations with CVD, as in the case with established risk factors, there was a strong reduction in absolute risk in each educational category after stratification (i.e. looking at the low-risk population without smoking, hypertension or hyperlipidemia), resulting in rather small changes in the relative gradient. With regard to the "modern" risk factor (here CRP), however, there were rather small changes in the absolute gradient after stratification (i.e., looking at subjects with CRP-levels below 3 mg/L), also resulting in rather small changes in the relative gradient. Even though, for several reasons, it is difficult to directly compare the effects of CRP and the established risk factors, it is essential to be aware of the fact that the interpretation of the importance of various risk factors in the association between SEP and CVD differs depending on which scale is used to measure social inequalities. These are important issues that affect opinions of where to invest effort in population-wide preventive programs and on the preventive potential of acting on reducing established risk factors as opposed to "modern" risk factors. The one-sided use of the relative scale can thus inhibit efforts to reduce established risk factors in that it gives priority to research and preventive programs looking in new directions.

## Competing interests

The authors declare that they have no competing interests.

## Authors' contributions

MR, GE and BH are responsible for the conception, design, analysis, and interpretation of the data; the drafting, writing, and revision of the content; and the approval of the final version. GB has made substantial contributions to the design and interpretation of the data, the revision of the content, and the approval of the final version.

## Pre-publication history

The pre-publication history for this paper can be accessed here:



## References

[B1] Kunst AE, Groenhof F, Mackenbach JP, Health EW (1998). Occupational class and cause specific mortality in middle aged men in 11 European countries: comparison of population based studies. EU Working Group on Socioeconomic Inequalities in Health. BMJ.

[B2] Labarthe D (1998). Epidemiology and prevention of cardiovascular diseases. A global challenge.

[B3] Tunstall-Pedoe H, Kuulasmaa K, Mähönen M, Tolenen H, Ruokoski E, Amoyel P (1999). Contribution of trends in survival and coronary-event rates to changes in coronary heart disease mortality: 10-year results from 37 WHO MONICA project populations. Monitoring trends and determinants in cardiovascular disease. Lancet.

[B4] Kaplan GA, Keil JE (1993). Socioeconomic factors and cardiovascular disease: a review of the literature. Circulation.

[B5] Nieto FJ (1999). Cardiovascular disease and risk factor epidemiology: a look back at the epidemic of the 20th century. Am J Public Health.

[B6] Huisman M, Kunst AE, Bopp M, Borgan J-K, Borrell C, Costa G, Deboosere P, Gadeyne S, Glickman M, Marinacci C, Minder C, Regidor E, Valkonen T, Mackenbach JP (2005). Educational inequalities in cause-specific mortality in middle-aged and older men and women in eight western European populations. Lancet.

[B7] Rothman K, Greenland S, Walker A (1980). Concepts of interaction. Am J Epidemiol.

[B8] Pearson TA, Mensah GA, Alexander RW, Anderson JL, Cannon RO, Criqui M, Fadl YY, Fortmann SP, Hong Y, Myers GL, Rifai N, Smith SC, Taubert K, Tracy RP, Vinicor F, Centers for Disease Control and Prevention; American Heart Association (2003). Markers of inflammation and cardiovascular disease: application to clinical and public health practice: A statement for healthcare professionals from the Centers for Disease Control and Prevention and the American Heart Association. Circulation.

[B9] Rosvall M, Engström G, Janzon L, Berglund G, Hedblad B (2007). The role of low grade inflammation as measured by C-reactive protein levels in the explanation of socioeconomic differences in carotid atherosclerosis. Eur J Public Health.

[B10] Magnus P, Beaglehole R (2001). The real contribution of the major risk factors to the coronary epidemics: time to end the "Only-50%" myth. Arch Intern Med.

[B11] Lynch J, Davey Smith G, Harper S, Bainbridge K (2006). Explaining the social gradient in coronary heart disease: comparing relative and absolute risk approaches. J Epidemiol Community Health.

[B12] Berglund G, Elmståhl S, Janzon L, Larsson SA (1993). The Malmö Diet and Cancer Study. Design and feasibility. J Int Med.

[B13] Manjer J, Carlsson S, Elmståhl S, Gullberg B, Janzon L, Lindström M, Mattisson I, Berglund G (2001). The Malmö diet and cancer study: representivity, cancer incidence and mortality in participants and non-participants. Eur J Cancer Prev.

[B14] Hedblad B, Nilsson P, Janzon L, Berglund G (2000). Relation between insulin resistance and carotid intima-media thickness and stenosis in non-diabetic subjects. Results from a cross-sectional study in Malmö, Sweden. Diabet Med.

[B15] Rosvall M, Östergren P-O, Hedblad B, Isacsson SO, Janzon L, Berglund G (2000). Occupational status, educational level and the prevalence of carotid atherosclerosis in a general population sample of middle-aged Swedish men and women. Results from the Malmö Diet and Cancer Study. Am J Epidemiol.

[B16] (1995). Treatment of Hyperlipidemia [in Swedish]. Information from the Medical Products Agency in Sweden.

[B17] Persson M, Hedblad B, Nelson JJ, Berglund G (2007). Elevated Lp-PLA2 levels add prognostic information to the metabolic syndrome on incidence of cardiovascular events among middle-aged nondiabetic subjects. Arterioscler Thromb Vasc Biol.

[B18] Anonymous (1997). Causes of Death 1995. Stockholm: The National Board of Health and Welfare, Centre of Epidemiology.

[B19] Tydén P, Hansen O, Janzon L (1998). Intra-urban variations in incidence and mortality in myocardial infarction. A study from the myocardial infarction register in the city of Malmö, Sweden. Eur Heart J.

[B20] (1997). World Health Organisation. International Classification of Diseases, Ninth Revision (ICD-9). Geneva: World Health Organisation.

[B21] Jerntorp P, Berglund G (1992). Stroke registry in Malmö, Sweden. Stroke.

[B22] Khan FA (2005). Epidemiology of stroke in an urban population – aspects of time, place and person. Thesis.

[B23] Li C, Engstrom G, Hedblad B, Berglund G, Janzon L (2005). Blood pressure control and risk of stroke: a population-based prospective cohort study. Stroke.

[B24] Syme SL (1996). Rethinking disease: where do we go from here?. Ann Epidemiol.

[B25] Boström G, Rosén M (2003). Measuring social inequalities in health – politics or science?. Scand J Public Health.

[B26] National Board of Health and Welfare (2001). Public Health Report of Sweden (in Swedish).

[B27] National Board of Health and Welfare (1997). Public Health Report of Sweden (in Swedish).

[B28] Hallqvist J, Lundberg M, Diderichsen F, Ahlbom A (1998). Socioeconomic differences in risk of myocardial infarction 1971-1994 in Sweden: time trends, relative risks and population attributable risks. Int J Epidemiol.

[B29] Danesh J, Wheeler JG, Hirschfield GM, Eda S, Eiriksdottir G, Rumley A, Lowe GD, Pepys MB, Gudnason V (2004). C-reactive protein and other circulating markers of inflammation in the prediction of coronary heart disease. N Engl J Med.

[B30] Lloyd-Jones DM, Tian L (2006). Predicting cardiovascular risk: so what do we do now?. Arch Intern Med.

[B31] Kivimäki M, Lawlor DA, Jounala M, Davey Smith G, Elovainio M, Keltikangas-Jarvinen L, Vahtera J, Viikari JS, Raitakari OT (2005). Lifecourse socioeconomic position, C-reactive protein, carotid intima-media thickness in young adults: the cardiovascular risk in Young Finns Study. Arterioscler Thromb Vasc Biol.

[B32] Wilson TW, Kaplan GA, Kauhanen J, Cohen RD, Wu M, Salonen R, Salonen JT (1993). Association between plasma fibrinogen concentration and five socioeconomic indices in the Koupio Ischemic Heart Disease Risk Factor Study. Am J Epidemiol.

[B33] Jousilahti P, Salomaa V, Rasi V, Vahtera E, Palosuo T (2003). Association of markers of systemic inflammation, C reactive protein, serum amyloid A, and fibrinogen, with socioeconomic position. J Epidemiol Community Health.

[B34] Owen N, Poulton T, Hay FC, Mohamed-Ali V, Steptoe A (2003). Socioeconomic status, C-reactive protein, immune factors, and responses to acute mental stress. Brain Behav Immun.

[B35] Lawlor DA, Smith GD, Rumley A, Lowe GD, Ebrahim S (2005). Associations of fibrinogen and C-reactive protein with prevalent and incident coronary heart disease are attenuated by adjustment for confounding factors. British Women's Heart and Health Study. Thromb Haemost.

[B36] Engstrom G, Hedblad B, Rosvall M, Janzon L, Lindgarde F (2006). Occupation, marital status, and low-grade inflammation. Mutual confounding or independent cardiovascular risk factors?. Arterioscler Thromb Vasc Biol.

[B37] Muennig P, Sohler N, Mahato B (2007). Socioeconomic status as an independent predictor of physiological biomarkers of cardiovascular disease: evidence from NHANES. Prev Med.

[B38] Engstrom G, Janzon L, Berglund G, Lind P, Stavenow L, Hedblad B, Lindgarde F (2002). Blood pressure increase and incidence of hypertension in relation to inflammation-sensitive plasma proteins. Arterioscler Thromb Vasc Biol.

[B39] Engstrom G, Hedblad B, Stavenow L, Lind P, Janzon L, Lindgarde F (2003). Inflammation-sensitive plasma proteins are associated with future weight gain. Diabetes.

[B40] Engstrom G, Hedblad B, Eriksson KF, Janzon L, Lindgarde F (2005). Complement C3 is a risk factor for the development of diabetes: a population-based cohort study. Diabetes.

[B41] Appelros P, Högerås N, Terent A (2003). Case ascertainment in stroke studies: the risk of selection bias. Acta Neurol Scand.

[B42] National Board of Health and Welfare Värdering av diagnoskvaliteten för akut hjärtinfarkt i patientregistret 1987 och 1995.

[B43] Winkleby MA, Jatulis DE, Frank E, Fortmann SP (1992). Socioeconomic status and health: How education, income and occupation contribute to risk factors for cardiovascular disease. Am J Public Health.

[B44] Lynch J, Kaplan G, Berkman L, Kawachi I (2000). Socioeconomic position. Social epidemiology.

[B45] Hallqvist J (1998). Socioeconomic differences in myocardial infarction risk. Epidemiological analyses of causes and mechanisms. (Thesis).

[B46] Kunst AE, Mackenbach JP (1994). The size of mortality differences associated with educational level in nine industrialized countries. Am J Public Health.

[B47] Rosvall M, Engström G, Hedblad B, Janzon L, Berglund G (2006). The role of preclinical atherosclerosis in the explanation of educational differences in incidence of coronary events. Atherosclerosis.

[B48] Vigushin DM, Pepys MB, Hawkins PN (1993). Metabolic and scintigraphic studies of radioiodinated human C-reactive protein in health and disease. J Clin Invest.

[B49] Rudnicka AR, Rumley A, Lowe GD, Strachan DP (2007). Diurnal, seasonal, and blood-processing patterns in levels of circulating fibrinogen, fibrin D-dimer, C-reactive protein, tissue plasminogen activator, and von Willebrand factor in a 45-year-old population. Circulation.

[B50] Meier-Ewert HK, Ridker PM, Rifai N, Price N, Dinges DF, Mullington JM (2001). Absence of diurnal variation of C-reactive protein concentrations in healthy human subjects. Clin Chem.

